# Predicting virological decay in patients starting combination antiretroviral therapy

**DOI:** 10.1097/QAD.0000000000001125

**Published:** 2016-06-29

**Keywords:** CD4^+^ cell count, combination antiretroviral therapy, HIV-1, predicted virological suppression, treatment switch, viral load

## Abstract

**Objective:**

Model trajectories of viral load measurements from time of starting combination antiretroviral therapy (cART), and use the model to predict whether patients will achieve suppressed viral load (≤200 copies/ml) within 6-months of starting cART.

**Design:**

Prospective cohort study including HIV-positive adults (UK Collaborative HIV Cohort Study).

**Methods:**

Eligible patients were antiretroviral naive and started cART after 1997. Random effects models were used to estimate viral load trends. Patients were randomly selected to form a validation dataset with those remaining used to fit the model. We evaluated predictions of suppression using indices of diagnostic test performance.

**Results:**

Of 9562 eligible patients 6435 were used to fit the model and 3127 for validation. Mean log_10_ viral load trajectories declined rapidly during the first 2 weeks post-cART, moderately between 2 weeks and 3 months, and more slowly thereafter. Higher pretreatment viral load predicted steeper declines, whereas older age, white ethnicity, and boosted protease inhibitor/non-nucleoside reverse transcriptase inhibitors based cART-regimen predicted a steeper decline from 3 months onwards. Specificity of predictions and the diagnostic odds ratio substantially improved when predictions were based on viral load measurements up to the 4-month visit compared with the 2 or 3-month visits. Diagnostic performance improved when suppression was defined by two consecutive suppressed viral loads compared with one.

**Conclusions:**

Viral load measurements can be used to predict if a patient will be suppressed by 6-month post-cART. Graphical presentations of this information could help clinicians decide the optimum time to switch treatment regimen during the first months of cART.

## Introduction

Combination antiretroviral therapy (cART) based on at least three antiretroviral drugs from at least two drug classes slows HIV replication and prevents transmission of HIV. Factors taken into consideration when selecting a patient's first cART-regimen include: the presence/absence of genotypic resistance against specific antiretroviral drugs; potential side-effects; comorbidities; drug interactions and patient preference [1]. Current guidelines recommend monitoring the effectiveness of first-line cART using routine viral load measurements (copies of HIV-1 RNA/millilitre of plasma) [1–3], at about 4-weeks after initiation of treatment and then every 3-months to confirm undetectable viral load levels [1].

HIV-dynamic studies have improved our understanding of the process of virus elimination after initiation of cART [4–5]. During the first few weeks of treatment there is a rapid decline in viral load, primarily because of the decay of productively infected cells [4,6–8]. The rate of decay becomes slower thereafter because of the release of HIV viruses by macrophages and other long-lived cells of the lymph nodes [4,5,8]. Finally, the decline levels off, probably because of reservoirs of long-lived cells still producing HIV virus [4]. In some cases the viral load level may rise again, for example, because of nonadherence to the cART regimen or emergence of resistant virus [4].

Clinicians may be tempted to increase monitoring or switch drug therapy during the phase of slow viral load decline, even though this is predictable and the patient is likely to achieve viral suppression. Early treatment switching may be unnecessary and has disadvantages, including that the new regimen may be less effective than the current one, a reduction in the number of available future treatment options, and the possibility of side-effects associated with the new regimen. Conversely, delays in switching regimen after virologic failure has occurred could result in the accumulation of resistance mutations, immunologic decline, and an increased risk of clinical events. Guidelines recommend that a switch of cART-regimen should be considered if a patient's viral load fails to fall to undetectable levels (<50 copies/ml) after 24–36 weeks of treatment [1,2].

In this article we model repeated measurements of viral load from start of cART to the first suppressed viral load. Among patients with at least two observed measurements, we use this model to predict a patient's future post-cART viral load measurements given their observed measurements up to 2,3, or 4 months post-cART. Based on these future measurements we predict whether patients will achieve a suppressed viral load measurement within 26-weeks of start of cART, test the reliability of these predictions, and show how this information can be used to enhance decisions on when to switch first-line cART.

## Methods

### Study patients

The UK Collaborative HIV Cohort study was initiated in 2001 and collates routine data on HIV-positive patients attending some of the largest clinical centres in the UK since 1 January 1996. The project was approved by a Multicentre Research Ethics committee and local ethics committees. Patients are included in the study provided they are HIV-positive, have attended one of the collaborating centres at any time since 1996 and are aged 16 years or over [9]. Analyses are based on data collected up to 31 December 2012.

Patients were eligible for analysis if they were antiretroviral naive, started cART after 1997, had at least one CD4^+^ measurement within the period 90 days before to 6 days after starting cART, at least one viral load measurement within the period 90 days before to 0 days after starting cART, and at least two post-cART viral load measurements observed within the first year of starting cART, where the first measurement was more than 200 copies/ml. Suppression was a priori defined as a single viral load 200 copies/ml or less.

### Statistical analyses

Because we were only concerned with modelling the viral decay phase from start of treatment to time of first suppression within the first year of cART, viral load measurements after time of first suppression or first year of cART were censored. Patients may stop or switch treatment regimens because of toxicities, side-effects, suspected treatment nonresponse, and other problems. Because stopping or switching treatment due to suspected treatment nonresponse could have biased our analyses and reasons for switching were sparsely recorded, we censored viral load measurements after a patient stopped treatment for at least 7 days or switched treatment. For a minority of patients their first suppressed viral load, included in the analysis, was below the detection limit and was replaced with the detection limit value.

Viral load measurements were log_10_ transformed to stabilize the variance and to meet normality assumptions of the residuals [10]. When modelling the relationship between log_10_-transformed viral load and time we considered a fractional polynomial of one and two degrees with powers −2, −1, −0.5, 0, 0.5, 1, 2, 3 (power zero is interpreted as a natural-log transformation) [11] and linear-spline models of one and two knots with the first knot at 2, 4, or 6 weeks and the second knot at 2, 3, or 4 months. We fitted random effects models with the intercept and trajectory terms random at the patient level, thus allowing viral load trajectories to vary between patients. We compared the fractional polynomials and linear spline models with respect to the Bayesian Information Criterion and satisfaction of the model's assumptions [12].

Patients were classified by their first-line cART regimen (non-nucleoside reverse transcriptase inhibitors (NNRTI-based), protease inhibitors (PI-based), boosted-PI, other), pretreatment CD4^+^ cell count (<25, 25–49, 50–99, 100–199, 200–349, 350–499, ≥500 cells/μl) and pretreatment viral load (<10 000, 10 000 to <100 000, 100 000 to <500 000, ≥500 000 copies/ml). Patients with more than one measurement within the pretreatment period were classified using the measurement closest to the start of cART.

We included covariates sex, age at start of cART, ethnicity, exposure, type of first-line cART regimen, pretreatment CD4^+^ cell count, and pretreatment viral load. For each covariate, interactions between the covariate and the intercept and trajectory terms were considered. We compared the Bayesian Information Criterion statistic of all models with up to five interactions.

Predictions of future viral-load measurements and the associated prediction error (the measure of uncertainty about those predictions) depend upon the fixed-effect coefficients and the variance parameters [13,14]. See Appendix for details about generation of these predictions and prediction error.

We validated the prediction model by randomly selecting patients to form a validation dataset. Because our aim was to predict suppression within the first 6 months of a patient starting (and continuing on) their first cART regimen, to form the validation dataset we randomly selected 40% of those patients who did not switch or stop treatment either before their first suppressed viral load or during the first 6 months since starting cART. The remaining patients (including those ineligible for random selection) formed the model-fitting dataset.

All patients in the model-fitting and validation datasets were used in the analysis to validate the prediction model. The model-fitting dataset was the training data for our prediction model. Using this model we predicted future viral load measurements for patients of the validation dataset. For patients in the model-fitting dataset we used all of their observed viral load measurements up to 1-year post-cART; and, for patients in the validation dataset we categorized viral load measurements within specific clinic visits by rounding the measurement time to the nearest month (e.g. measurements at 2.7 and 3.12 months were categorized as observed at the 3-month visit). Observed viral load measurements up to and including specified clinic visits were used to predict future measurements. We only predicted future measurements among patients who were not censored (because of suppression, treatment switching, or dropout) at the follow-up prior to the time interval being predicted.

Based on the predicted future viral load trajectories we predicted whether each patient would achieve suppression (single predicted viral load ≤200 copies/ml) within 6 months of starting cART. We also classified patients in the validation dataset according to whether they were observed to achieve suppression (single observed viral load ≤ 200 copies/ml) within 6 months of starting cART. We evaluated prediction of suppression using common indices of diagnostic test performance: sensitivity, specificity, positive-predictive value, negative-predictive value, likelihood-ratio of a positive result, likelihood-ratio of a negative result and the diagnostic odds ratio [15]. We conducted four sensitivity analyses: suppression defined by two consecutive viral load measurements ≤200 copies/ml, patients of the validation dataset randomly selected from all eligible patients, viral load measurements not censored after a patient stopped or switched treatment, and among the first suppressed viral load measurements we censored those measurements below the detection limit.

Following Taylor, Yu, and Sandler [16], we derived prediction graphs depicting patients’ predicted viral load measurements (with 95% prediction intervals) up to 6 months post-cART, patients’ observed measurements from previous visits and their measurement from the current visit. Using this most recent measurement, a new graph can be produced, allowing real time monitoring of patients’ progression.

## Results

Of 47 201 patients included in UK Collaborative HIV Cohort study up to 31 December 2012, 24 135 started cART before 1998 or before entering the study, or did not start cART. A further 5235 had no CD4 or viral load measurements within the specified pretreatment periods. Of the remaining patients, 1617 were suppressed before start of cART, 519 had zero post-cART viral load measurements, 385 had one (unsuppressed) post-cART viral load measurement, and for 5748 their first post-cART viral load measurement was suppressed, leaving 9562 eligible for analyses. Table 1 presents patient characteristics according to pretreatment viral load. Most were men, approximately half were homosexual or bisexual, of white ethnicity and started on a NNRTI-based cART regimen. Compared with patients with pretreatment viral load of at least 10 000 copies/ml, a higher proportion of patients with pretreatment viral load less than 10 000 copies/ml were women, Black African, heterosexual, and started on a boosted-PI cART regimen. Median pretreatment CD4^+^ decreased with increasing pretreatment viral load.

A total of 7249 (76%) patients achieved at least one suppressed viral load measurement within the first year of cART. Among these, the median time to first suppressed viral load measurement was 2.76 [interquartile range (IQR) 1.91–3.91] months and the median number of viral load measurements, up to and including the first suppressed measurement, was 4 [IQR 3–5] measurements. Of the 2313 (24%) patients who did not achieve at least one suppressed viral load, the median number of viral load measurements was 3 [IQR 2–4].

**Fig. 1:**
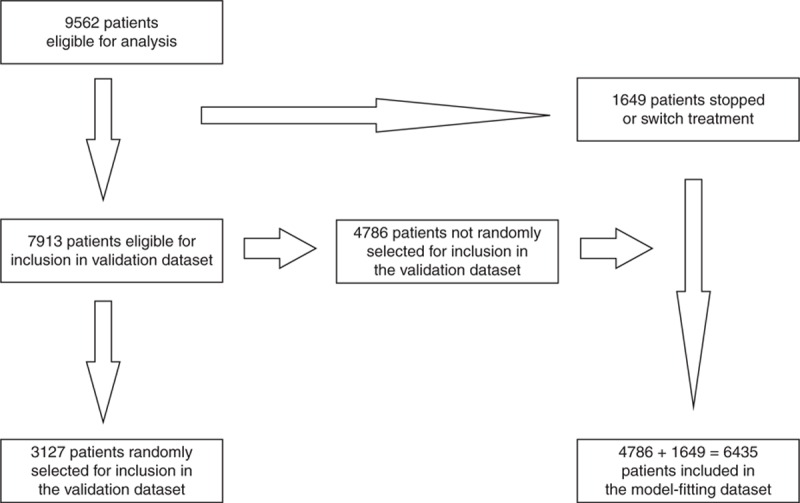
A flowchart depicting assignment of the patients eligible for analysis to the validation and model-fitting datasets.

Among the 9562 patients eligible for analysis, 1649 (17%) stopped their first-line cART regimen (for at least 7 days) or switched to a second-line cART regimen either before their first suppressed viral load or during the first 6 months after starting cART. We randomly selected 3127 (40%) of the remaining 7913 patients to form the validation dataset. The 6435 patients not randomly selected (including the 1649 ineligible for random selection) formed the model-fitting dataset. Figure 1 shows how the patients eligible for analysis were assigned to the validation and model-fitting datasets. The patients’ characteristics in the model-fitting (Appendix-table 2) and validation (Appendix-table 3) datasets were similar.

**Fig. 2:**
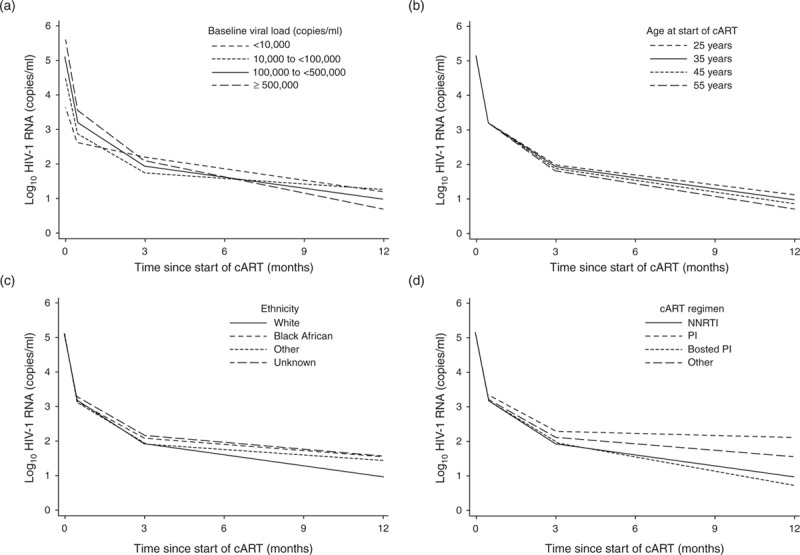
Predicted mean log_10_ HIV-1 RNA trajectories within the first year of starting combination cART according to (a) baseline viral load groups, (b) age at start of cART, (c) ethnic group and (d) type of cART-regimen. cART, combination antiretroviral therapy.

Figure 2 shows mean log_10_ viral load trajectories predicted by the best fitting model, a linear spline with knots at 2 weeks and 3 months post-cART, in which mean log_10_ viral load trajectories varied between patients with different pretreatment viral load group, age at start of cART, ethnic group, and type of first-line cART regimen. For all patient groups except those with pretreatment viral load less than 10 000 copies/ml, mean log_10_ viral load trajectories declined rapidly between start of cART and 2 weeks post-cART, moderately between 2 weeks and 3 months and more slowly from 3 months onwards. Higher pretreatment viral load predicted a steeper decline in mean log_10_ viral load for all three phases. For example, among patients with pretreatment viral load between 10 000 and less than 100 000 copies/ml estimated decline in mean log_10_ viral load during phases 1,2, and 3 were respectively 3.58 [95% CI 3.52, 3.65], 0.39 [95% CI 0.36, 0.41] and 0.06 [95% CI 0.03, 0.08] log_10_ copies/ml/month, whereas among patients with pretreatment viral load of at least 500 000 copies/ml the corresponding declines were 4.46 [95% CI 4.38, 4.54], 0.56 [95% CI 0.53, 0.59], and 0.15 [95% CI 0.12, 0.17] log_10_ copies/ml/month. For the first and second phases there was little difference according to age and ethnic group, and the decline of mean log_10_ viral load was more gradual for PI-based regimen than for the other cART-regimen groups. During the third phase, older age at start of cART predicted a steeper decline, the decline was steeper for White than non-White patients, and steeper for boosted-PI and NNRTI-based regimens than for PI-based or other regimens.

The solid black line in each graph denotes the predicted mean log_10_ HIV-1 RNA trajectory for the reference patient: white men, aged 35 years at start of cART, homosexual or bisexual, first-line cART-regimen includes a NNRTI, pretreatment CD4^+^ cell count between 200 and 349 cells/μl and pretreatment viral load between 100 000 and less than 500 000 copies/ml.

Table 2 compares observed and predicted viral suppression within 6-months of start of cART among patients in the validation dataset, based on observed viral load measurements up to and including the 2, 3, and 4-month visits. Because predictions were not generated for patients who were censored on or before the specified visit or who did not have an observed measurement at the specified visit, the number of patients in the validation dataset decreases from the 2-month to the 4-month visit. Between the 2 and 4-month visits, specificity of the predictions substantially improved whereas sensitivity of the predictions slightly decreased. Also diagnostic accuracy improved substantially, from diagnostic odds ratio 5.25 [95% CI 4.09, 6.74] at 2 months to 15.60 [10.77, 22.56] at 4 months.

Compared with suppression defined by a single viral load 200 copies/ml or less, under the stricter definition of suppression based on two consecutive viral loads 200 copies/ml or less then, at each specified visit, the number of patients at risk (i.e. not previously suppressed) was higher and the percentage of patients observed and predicted to be suppressed was lower (Appendix-table 4). Specificity and negative-predictive value were substantially higher under the stricter definition of suppression. All indicators of diagnostic performance showed greater accuracy of predicting suppression when suppression was defined by two consecutive viral loads of 200 copies/ml or less compared with a single viral load 200 copies/ml or less.

The results of the remaining sensitivity analyses, where: the validation dataset was a random sample of all patients eligible for analysis (Appendix-table 5), measurements after stopping or switching treatment were not censored (Appendix-table 6), and first suppressed viral loads below the detection limit were censored (Appendix-table 7), were similar to the results of the main analysis (Table 2).

### Predicting time to suppression

**Fig. 3:**
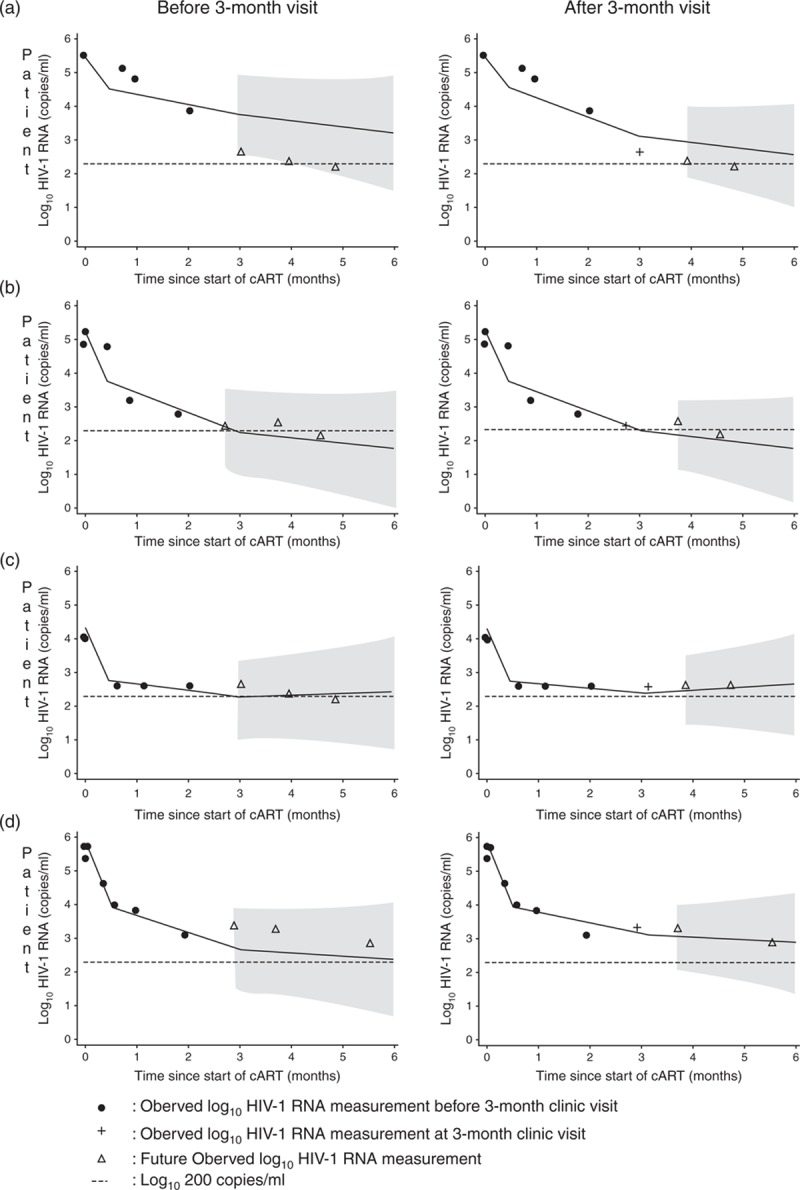
Prediction graphs of four selected patients based on observations measured before 3-month visit (left-hand column) and on observations measured after 3-month visit (right-hand column).

Figure 3 compares observed with predicted future viral load measurements before and after the 3-month visit, for patients who were selected to illustrate a range of viral load patterns and predictions. The shaded areas denote 95% prediction intervals for each patient. Because patients had a small number of observed measurements the prediction intervals were wide.

The solid line is the patient's predicted log_10_ HIV-1 RNA trajectory with 95% uncertainty intervals (shaded regions). The dashed line indicates the cut-off for suppression (200 copies/ml).

At the 3-month visit patient-A was not predicted to achieve suppression within 6 months of starting cART (left-hand graph). The new measurement (labelled +) was better than expected (below the predicted trajectory) and the updated graph predicted a steeper decline from 3 to 6 months (right-hand graph), although still not predicted to be suppressed by 6 months. Patient-B was predicted to be suppressed approximately 3 months post-cART (left-hand graph) and the new measurement agrees with the predicted trajectory, and so very little has changed in the updated prediction (right-hand graph). Based on these graphs, a clinician may decide that patients A and B should continue on their first-line cART regimen, as they are predicted to decline steadily, and to next measure the patients’ viral load at the 5-month visit to confirm that they have become suppressed. Patient-C was initially predicted to achieve suppression by 3 months post-cART and patient-D was predicted to steadily decline almost achieving suppression by 6 months. Their 3-month measurements were worse than expected (above the predicted trajectory) and the updated graphs show that they were less likely to be suppressed by 6 months, which is consistent with their future measurements. For patient-C a clinician may decide at the 3-month visit to switch to second-line cART therapy as the patient's trajectory is predicted to level off to above 200 copies/ml. For patient-D a clinician may decide to continue with the first-line cART therapy and to measure the patient's viral load at 4 months post-cART to confirm that the decline has slowed down. The clinician could then update the prediction graph using the 4-month measurement and review the decision to maintain the first-line regimen.

## Discussion

We fitted a flexible linear mixed-effects model to repeated viral load measurements from the time of starting cART, and used this model to predict the effectiveness of the first cART regimen in achieving viral load suppression based on individual patients’ pretreatment clinical information and post-cART viral load measurements. Mean log_10_ viral load trajectories declined rapidly between start of cART and 2 weeks post-cART, moderately between 2 weeks and 3 months and more slowly thereafter. Higher pretreatment viral load predicted a steeper decline in mean log_10_ viral load for all three phases. During the third phase, older age at start of cART predicted a steeper decline, the decline was steeper for White than non-White patients, and steeper for boosted-PI and NNRTI-based regimens than for PI-based or other regimens. The model's predictive ability improved markedly when based on viral load measurements up to the 4-month clinic visit compared with the 2 or 3-month visits. Patients’ current viral load trajectory and future viral load predictions can be graphically presented and used to assess if a patient is likely to become virologically suppressed within 6 months of start of treatment whilst on their current regimen.

Among the patients eligible for analysis 60% (5753) had at least one post-cART viral load within the first 2 weeks since starting treatment and so we are confident that our data support estimation of a change in viral load within the first 2 weeks. A key feature is that the model predicts future viral load measurements using a series of observed measurements, making efficient use of all available data. Furthermore, the predictions can be updated as new measures are obtained, which further improves prediction accuracy.

This study has several limitations. Patients’ measurements were censored after the first occurrence of a suppressed viral load measurement and so those patients who had a rapid decline in viral load contribute only a few observations to the model. Our model cannot reliably predict suppression before 3 months post-cART, which occurred among 3187 (33%) of the patients eligible for inclusion in our analyses. Only a few patients were treated with integrase inhibitors, which are now more widely used. Our predictions were based on a small number of observed measurements: the prediction intervals were consequently wide. Some patients stopped taking treatment or switched to a second-line cART regimen before their viral load measurements had dropped below 200 copies/ml. Information on reasons for a change in treatment was not available. We censored all viral load measurements that were observed after a patient stopped or switched treatment and, in a sensitivity analysis, inclusion of these censored measurements did not change our conclusions. Lastly, patients may have dropped out of the study because of reasons unrelated to virological response, or because of loss to follow-up or AIDS-related mortality. Random effects models, as used in this study, are robust to dropout that is predictable from observed data (‘missing at random’) [17,18] but our estimates may have been biased by a dropout mechanism that is not predicted by observed viral load measurements.

Several HIV-dynamic studies, modelling data from start of treatment up to 8 or 12-weeks posttreatment, have reported a rapid decline in weeks 1–3 and a slower decline thereafter [19–28]. A HIV-dynamic study with 72-weeks of follow-up reported three phases of decreasing decay rates, where the transition from phases 1–2 was estimated at 16.1 days and from phases 2–3 at 15.7 weeks [29]. A cohort of cART-naive and cART-experienced patients, with measurements at 2 weeks, 3, 6, and 9 months, modelled viral decay using a linear spline with a single knot at 3 months [30].

Our finding that higher pretreatment viral load predicted steeper declines in mean log_10_ viral load is broadly consistent with the literature [19,21,28,31]. Findings in some studies that trends did not differ by pretreatment viral load [20], or that higher pretreatment viral load predicted slower decline during phase-1 [22,26], may be explained by differences in the potency of the treatment regimens and pretreatment virus clearance ratios and turnover rates of infected cells [21]. Although a few small studies (<225 patients) reported that viral load trends did not differ by age or ethnicity [19,22,30], our findings that older age predicted steeper declines and that declines were steeper for White than non-White patients are consistent with reports that older age predicted a shorter time to suppression [32–37] and that White patients are more likely to become suppressed than non-Whites [37–43]. In keeping with our results Wu *et al.*[21] reported a steeper decline for NNRTI-based regimens compared with a PI-based regimen.

Several studies have reported that declines in viral load during weeks 1–3 predicted virological response at 8, 12 and 24 weeks [19,23,24,27] and that viral load measurements at 4 and 8 weeks were strong predictors of virological response at 24 weeks [44,45]. However, our study is the first of which we are aware to use all available viral load measurements to predict first suppression by 24 weeks.

We have shown that frequent viral load monitoring can reliably predict by 4 months post-cART if a patient will be suppressed within 6 months of starting treatment. Presenting the observed and future predicted measurements in a graphical plot could aid clinicians in their decision whether to change cART regimens in patients not suppressed by 3 months post-cART. Possible actions might include: returning at 6 months post-cART to confirm viral load suppression, returning in 1 month for next viral load measurement to minimize any uncertainty, or switch to second-line therapy. We hope that the information provided in these prediction graphs will provide reassurance in making robust decisions regarding future cART regimens, and avoid unnecessary changes of regimen.

In conclusion, we have shown how a series of viral load measurements can be utilized to predict future viral load measurements, and how this information can be presented graphically. Future work could extend models to allow for informative dropout and develop a web-based tool [46], where a clinician inputs the information into a web-based calculator and the tool outputs a prediction graph.

## Acknowledgements

The authors would like to thank all the clinicians, data managers, and research nurses in participating clinical centres who have assisted with the provision of data for this project.

Writing committee: Rachael A. Hughes^a^, Jonathan A.C. Sterne^a,b^, John WALSH^c^, Frank POST^d^, Mark Nelson^e^, Sophie Jose^f^, Teresa Hill^f^, Kate Tilling^a^, Martin Fisherg^∗^, David Dunn^h^, Roy TREVELION^i^, Adrian Palfreeman^j^, Fabiola Martin^k^, Mark Gompels^l^, Clifford LEEN^m^, Margaret Johnson^n^; Richard Gilson^o^, Jane Anderson^p^; Jonathan Ainsworth^q^; Phillip Hay^r^, Chloe Orkin^s^ Stephen Kegg^t^, Caroline A. Sabin^f,u^.

^a^School of Social and Community Medicine, University of Bristol, Bristol; ^b^The National Institute for Health Research Health Protection Research Unit (NIHR HPRU) in Evaluation of Interventions at University of Bristol; ^c^Imperial College Healthcare NHS Trust, London; ^d^King's College Hospital NHS Foundation Trust, HIV Research Centre, London; ^e^Chelsea and Westminster Hospital, London; ^f^Research Department of Infection and Population Health, University College London, London; ^g^Brighton and Sussex University Hospitals NHS Trust, Brighton; ^h^MRC Clinical Trials Unit at University College London, London; ^i^UK Community Advisory Board; ^j^Leicester Royal Infirmary, Leicester; ^k^York Teaching Hospital NHS Foundation Trust, York; ^l^North Bristol NHS Trust, Bristol; ^m^The Lothian University Hospitals NHS Trust, Edinburgh; ^n^Royal Free Hampstead NHS Trust, London; ^o^Mortimer Market Centre, University College Medical School, London; ^p^Homerton University Hospital NHS Trust, London; ^q^North Middlesex University Hospital NHS Trust, London; ^r^St George's Healthcare NHS Trust, London; ^s^Barts Health NHS Trust, London; ^t^South London Healthcare NHS Trust, London; ^u^The National Institute for Health Research Health Protection Research Unit (NIHR HPRU) in Blood Borne and Sexually Transmitted Infections at University College London, UK

Steering committee: Jonathan Ainsworth, Sris Allan, Jane Anderson, Abdel Babiker, David Chadwick, Valerie Delpech, David Dunn, Martin Fisher^∗^, Brian Gazzard, Richard Gilson, Mark Gompels, Phillip Hay, Teresa Hill, Margaret Johnson, Sophie Jose, Stephen Kegg, Clifford Leen, Fabiola Martin, Mark Nelson, Chloe Orkin, Adrian Palfreeman, Andrew Phillips, Deenan Pillay, Frank Post, Jillian Pritchard, Caroline Sabin, Memory Sachikonye, Achim Schwenk, Anjum Tariq, John Walsh.

Central coordination: University College London (Teresa Hill, Sophie Jose, Andrew Phillips, Caroline Sabin, Alicia Thornton); Medical Research Council Clinical Trials Unit at UCL (MRC CTU at UCL), London (David Dunn, Adam Glabay).

Participating centres: Brighton and Sussex University Hospitals NHS Trust (Martin Fisher^∗^, Nicky Perry, Stuart Tilbury, Elaney Youssef, Duncan Churchill); Chelsea and Westminster Hospital NHS Foundation Trust, London (Brian Gazzard, Mark Nelson, Rhiannon Everett, David Asboe, Sundhiya Mandalia); King's College Hospital NHS Foundation Trust, London (Frank Post, Hardik Korat, Chris Taylor, Zachary Gleisner, Fowzia Ibrahim, Lucy Campbell); Mortimer Market Centre, University College London (Richard Gilson, Nataliya Brima, Ian Williams); Royal Free NHS Foundation Trust/University College London (Margaret Johnson, Mike Youle, Fiona Lampe, Colette Smith, Rob Tsintas, Clinton Chaloner, Samantha Hutchinson, Caroline Sabin, Andrew Phillips Teresa Hill, Sophie Jose, Alicia Thornton, Susie Huntington); Imperial College Healthcare NHS Trust, London (John Walsh, Nicky Mackie, Alan Winston, Jonathan Weber, Farhan Ramzan, Mark Carder); Barts and The London NHS Trust, London (Chloe Orkin, Janet Lynch, James Hand, Carl de Souza); Homerton University Hospital NHS Trust, London (Jane Anderson, Sajid Munshi); North Middlesex University Hospital NHS Trust, London (Jonathan Ainsworth, Achim Schwenk, Sheila Miller, Chris Wood); The Lothian University Hospitals NHS Trust, Edinburgh (Clifford Leen, Alan Wilson, Sheila Morris); North Bristol NHS Trust (Mark Gompels, Sue Allan); Leicester, University Hospitals of Leicester NHS Trust (Adrian Palfreeman, Khurram Memon, Adam Lewszuk); Middlesbrough, South Tees Hospitals NHS Foundation Trust, (David Chadwick, Emma Cope, Jane Gibson); Woolwich, Lewisham and Greenwich NHS Trust (Stephen Kegg, Paul Main, Dr Mitchell, Dr Hunter), St. George's Healthcare NHS Trust (Phillip Hay, Mandip Dhillon); York Teaching Hospital NHS Foundation Trust (Fabiola Martin, Sarah Russell-Sharpe); Coventry, University Hospitals Coventry and Warwickshire NHS Trust (Sris Allan, Andrew Harte, Stephen Clay); Wolverhampton, The Royal Wolverhampton Hospitals NHS Trust (Anjum Tariq, Hazel Spencer, Ron Jones); Chertsey, Ashford and St.Peter's Hospitals NHS Foundation Trust (Jillian Pritchard, Shirley Cumming, Claire Atkinson); Public Health England, London (Valerie Delpech); UK Community Advisory Board (Roy Trevelion).

^∗^Deceased.

R.A.H. was supported by Medical Research Council grant [MR/J013773/1]. J.A.C.S. was supported by grant number MR/J002380/1: this award was jointly funded by the UK Medical Research Council (MRC) and the UK Department for International Development (DFID) under the MRC/DFID Concordat agreement and is also part of the EDCTP2 programme supported by the European Union. He was also supported by the National Institute for Health Research Senior Investigator award NF-SI-0611-10168. The UK CHIC Study is funded by the Medical Research Council (MRC) UK (grant numbers G0000199, G0600337, G0900274 and M004236). The views expressed in this manuscript are those of the researchers and not necessarily those of the MRC.

This report is independent research supported by the National Institute for Health Research. The research was funded by the National Institute for Health Research Health Protection Research Units (NIHR HPRUs) in Blood Borne and Sexually Transmitted Infections at UCL and in collaboration with the London School of Hygiene and Tropical Medicine, and in Evaluation of Interventions at University of Bristol, both of which are in partnership with Public Health England. The views expressed in this publication are those of the author(s) and are not necessarily those of the NHS, the National Institute for Health Research, the Department of Health or Public Health England.

J.W. proposed the project. C.A.S. and J.A.C.S. designed the study. R.A.H. carried out the statistical analysis with participation from J.A.C.S., K.T. and C.A.S. R.A.H., J.A.C.S., C.A.S. and K.T. drafted the manuscript. All other authors contributed to the study design and data collection, and participated in the manuscript preparation. All authors reviewed and approved the final version of the manuscript.

### Conflicts of interest

There are no conflicts of interest.

## Supplementary Material

Supplemental Digital Content

## Figures and Tables

**Table 1:** Characteristics of the 9562 eligible patients.

	Pretreatment HIV-1 RNA (copies/ml)			
	<10 k^b^	10 k to <100 k	100 k to <500 k	≥500 k
Number of patients	756	3372	3825	1609
Median (IQR)^a^ age (years)	36 (31–42)	37 (31–43)	37 (32–44)	38 (33–45)
Male (%)	56	74	79	79
Risk group (%)				
Homo/bisexual	35	55	61	59
IDU	4	2	2	2
Heterosexual	55	37	32	35
Other/not known	6	5	4	4
Ethnicity (%)				
White	40	57	61	62
Black African	43	27	23	25
Other	14	14	14	12
Not known	3	2	2	1
First-line cART-regimen (%)				
NNRTI-based	52	63	67	63
PI-based	8	5	5	5
Boosted-PI	33	27	23	27
Other	7	5	5	5
Median (IQR) pretreatment HIV-1 RNA (log_10_ copies/ml)	3.43 (2.86–3.78)	4.67 (4.43–4.86)	5.32 (5.15–5.50)	5.88 (5.71–6.00)
Median (IQR) pretreatment CD4^+^ cell count (cells/μl)	272 (180–400)	236 (159–320)	180 (84–270)	114 (42–218)

^a^IQR, Inter quartile range.

^b^k, A thousand. cART, combination antiretroviral therapy.

**Table 2:** Validation of the model for predicting future suppression by 6 months since start of treatment given observations up to a specified visit.

	2-month visit	3-month visit	4-month visit
Number patients^a^	1927	1127	698
Observed suppressed	81%	69%	51%
Predicted suppressed	80%	67%	51%
Sensitivity [95% CI]	86% [84%, 88%]	81% [79%, 84%]	80% [76%, 85%]
Specificity [95% CI]	46% [41%, 51%]	63% [58%, 68%]	79% [75%, 83%]
PPV [95% CI]	87% [85%, 89%]	83% [80%, 86%]	80% [76%, 84%]
NPV [95% CI]	44% [39%, 49%]	60% [55%, 65%]	79% [75%, 84%]
LR+ [95% CI]	1.60 [1.45, 1.76]	2.21 [1.92, 2.55]	3.86 [3.12, 4.78]
LR− [95% CI]	0.30 [0.26, 0.36]	0.30 [0.25, 0.35]	0.25 [0.20, 0.31]
DOR [95% CI]	5.25 [4.09, 6.74]	7.49 [5.65, 9.93]	15.60 [10.77, 22.56]

^a^Number of patients not suppressed at the specified visit and with at least one future measurement. CI, confidence interval; DOR, diagnostic odds-ratio; LR−, likelihood ratio of a negative result; LR+, likelihood ratio of a positive result; NPV, negative predictive value; PPV, positive predictive value.
