# Supplementary material for: Predicting virological decay in patients starting combination antiretroviral therapy
Source: AIDS. 2016 Jun 29;30(11):1817–27. doi: 10.1097/QAD.0000000000001125 (PMC4933580; doi:10.1097/QAD.0000000000001125)
Supplement: Supplemental Digital Content [file aids-30-1817-s001.docx]

**Appendix**

We used the parameter estimates from our final random-effects model (Appendix-table 1) to generate predictions of future viral load (VL) measurements and the associated prediction error. Following Taylor and Law, we describe how these predictions were generated for patient $i$[1].

Suppose patient $i$ has $n_{i}$ observed VL measurements $Y_{i}=\left( Y_{i1},\cdots Y_{ij}, \cdots,Y_{in_{i}} \right)$, where $Y_{ij}$ is the log_10_ VL measurement observed at measurement time-point $j$. The random-effects model is

$Y_{i}=X_{i}\beta+Z_{i}u_{i}+e_{i}$,

with fixed-effects coefficients $\beta$ and design matrix $X_{i}$, random-effects coefficients $u_{i}$ and design matrix $Z_{i}$ and level-1 residuals $e_{i}$. The random effects $u_{i}$ and residuals $e_{i}$ are independently, normally distributed with zero means and covariances $G$ and $\sigma^{2}I_{n_{i}}$.

We wish to predict $n_{i}^{F}$future log_10_ VL measurements $Y_{i}^{F}$ at pre-specified time-points. Let $X_{i}^{F}$and $Z_{i}^{F}$denote the fixed-effects and random-effects design matrices corresponding to these future time-points. To generate the predictions we require the following components: $\Omega_{i}=Z_{i}G\left( Z_{i} \right)^{T}+\sigma^{2}I_{n_{i}}$, $\Lambda_{i}=Z_{i}^{F}G\left( Z_{i} \right)^{T}$ and $\Omega_{i}^{F}=Z_{i}^{F}G\left( Z_{i}^{F} \right)^{T}+\sigma^{2}I_{n_{i}}^{F}$. The prediction of future measurements $Y_{i}^{F}$given the observed measurements $Y_{i}$ is

$$Y_{i}^{F}=X_{i}^{F}\beta+\Lambda_{i}^{F}\left( \Omega_{i} \right)^{-1}\left( Y_{i}-X_{i}\beta\right)$$

and the prediction error is

$\Omega_{i}^{F}-\Lambda_{i}^{F}\left( \Omega_{i} \right)^{-1}\left( \Lambda_{i}^{F} \right)^{T}+\left( X_{i}^{F}-\Lambda_{i}^{F}\left( \Omega_{i} \right)^{-1}X_{i} \right)var\left( \beta\right)\left( X_{i}^{F}-\Lambda_{i}^{F}\left( \Omega_{i} \right)^{-1}X_{i} \right)^{T}$,

where $var\left( \beta\right)$ represents the covariance matrix of the fixed-effects coefficients $\beta$ and is obtained from the fitted random-effects model.

Our approximation for the prediction error accounted for uncertainty in the estimation of the fixed effects coefficients and variance parameters, but not the estimation of the variances and covariances between the observed and future measurements[1]. Provided the sample size is reasonably large then this uncertainty can be ignored[1,2].

Appendix-table 1: Coefficients of the final model

| Fixed effects | |  | Estimates reported on the log_10_ scale  [95% confidence interval] |
| --- | --- | --- | --- |
| Reference group^a^ | | Constant | 5.11 [5.10, 5.13] |
|  | | Time^b^: 0 to 2 weeks | -4.18 [-4.24, -4.12] |
|  | | Time: 2 weeks to 3 months | -0.49 [-0.51, -0.47] |
|  | | Time: 3 to 12 months | -0.10 [-0.08, -0.13] |
|  | |  |  |
|  | Compared to the reference group | |  |
|  | IDU | Constant | 0.03 [-0.02, 0.07] |
|  | Heterosexual | Constant | -0.01 [-0.03, 0.02] |
|  | Other risk group | Constant | 0.01 [-0.03, 0.04] |
|  |  |  |  |
|  | CD4^c^ < 25 | Constant | 0.12 [0.09, 0.15] |
|  | CD4 25 to 49 | Constant | 0.11 [0.07, 0.14] |
|  | CD4 50 to 99 | Constant | 0.10 [0.08, 0.12] |
|  | CD4 100 to 199 | Constant | 0.05 [0.03, 0.07] |
|  | CD4 350 to 500 | Constant | -0.02 [-0.04, 0.01] |
|  | CD4 500 | Constant | 0.003 [-0.03, 0.04] |
|  |  |  |  |
|  | VL^d^ < 10000 | Constant | -1.48 [-1.51, -1.45] |
|  |  | Time: 0 to 2 weeks | 1.91 [1.78, 2.04] |
|  |  | Time: 2 weeks to 3 months | 0.35 [0.31, 0.39] |
|  |  | Time: 3 to 12 months | -0.02 [-0.06, 0.02] |
|  |  |  |  |
|  | VL 10000 to < 100000 | Constant | -0.60 [-0.62, -0.58] |
|  |  | Time: 0 to 2 weeks | 0.60 [0.53, 0.67] |
|  |  | Time: 2 weeks to 3 months | 0.06 [0.03, 0.08] |
|  |  | Time: 3 to 12 months | 0.04 [0.01, 0.07] |
|  |  |  |  |
|  | VL 500000 | Constant | 0.50 [0.48, 0.52] |
|  |  | Time: 0 to 2 weeks | -0.32 [-0.41, -0.24] |
|  |  | Time: 2 weeks to 3 months | -0.08 [-0.11, -0.06] |
|  |  | Time: 3 to 12 months | -0.05 [-0.09, -0.02] |
|  |  |  |  |
|  | Age at start of cART | Constant | -0.001 [0.00, 0.002] |
|  |  | Time: 0 to 2 weeks | -0.0004 [-0.004, 0.003] |
|  |  | Time: 2 weeks to 3 months | -0.003 [-0.004, -0.002] |
|  |  | Time: 3 to 12 months | -0.001 [-0.002, 0.0005] |
|  |  |  |  |
|  | Black African | Constant | -0.04 [-0.06, -0.01] |
|  |  | Time: 0 to 2 weeks | -0.06 [-0.13, 0.01] |
|  |  | Time: 2 weeks to 3 months | 0.09 [0.06, 0.11] |
|  |  | Time: 3 to 12 months | 0.04 [0.02, 0.07] |
|  |  |  |  |
|  | Other ethnicity | Constant | -0.02 [-0.04, 0.001] |
|  |  | Time: 0 to 2 weeks | -0.08 [-0.17, 0.004] |
|  |  | Time: 2 weeks to 3 months | 0.03 [-0.002, 0.06] |
|  |  | Time: 3 to 12 months | 0.04 [0.004, 0.08] |
|  |  |  |  |
|  |  |  |  |
|  |  |  |  |
|  | Appendix-table 1 continued: Coefficients of the final model | | |
|  | Fixed effects |  | Estimates reported on the log_10_ scale  [95% confidence interval] |
|  | Unknown ethnicity | Constant | -0.01 [-0.06, 0.05] |
|  |  | Time: 0 to 2 weeks | 0.25 [0.03, 0.47] |
|  |  | Time: 2 weeks to 3 months | 0.07 [-0.002, 0.14] |
|  |  | Time: 3 to 12 months | 0.03 [-0.06, 0.11] |
|  |  |  |  |
|  | PI-based regimen | Constant | -0.02 [-0.05, 0.01] |
|  |  | Time: 0 to 2 weeks | 0.37 [0.22, 0.51] |
|  |  | Time: 2 weeks to 3 months | 0.09 [0.04, 0.14] |
|  |  | Time: 3 to 12 months | 0.08 [0.04, 0.12] |
|  |  | |  |
|  | Boosted PI-based | Constant | 0.02 [0.002, 0.03] |
|  | Regimen | Time: 0 to 2 weeks | 0.31 [0.24, 0.38] |
|  |  | Time: 2 weeks to 3 months | -0.05 [-0.07, -0.02] |
|  |  | Time: 3 to 12 months | -0.03 [-0.06, -0.001] |
|  |  |  |  |
|  | Other regimen | Constant | -0.05 [-0.08, -0.02] |
|  |  | Time: 0 to 2 weeks | 0.22 [0.08, 0.35] |
|  |  | Time: 2 weeks to 3 months | 0.06 [0.01, 0.10] |
|  |  | Time: 3 to 12 months | 0.04 [-0.01, 0.09] |
|  | |  |  |
| Random effects | |  | Variance or covariance  [95% confidence interval] |
|  | Individual level |  |  |
|  | Constant | | 0.036 [0.033, 0.039] |
|  | 0 to 2 weeks | | 0.624 [0.566, 0.689] |
|  | 2 weeks to 3 months | | 0.067 [0.061, 0.073] |
|  | 3 to 12 months | | 0.039 [0.034, 0.044] |
|  | Constant, 0 to 2 weeks | | 0.034 [0.024, 0.043] |
|  | Constant, 2 weeks to 3 months | | -0.008 [-0.012, -0.005] |
|  | Constant, 3 to 12 months | | -0.005 [-0.009, -0.001] |
|  | 0 to 2 weeks, 2 weeks to 3 months | | 0.041 [0.026, 0.056] |
|  | 0 to 2 weeks, 3 to 12 months | | -0.070 [-0.087, -0.054] |
|  | 2 weeks to 3 months, 3 to 12 months | | 0.002 [-0.003, 0.006] |
|  |  | |  |
|  | Measurement level |  |  |
| Constant | | | 0.159 [0.155, 0.162] |

^a^ Reference patient: white male, aged 35 years at start of cART, homosexual or bisexual, first-line cART-regimen includes a NNRTI, pre-treatment CD4 count between 200 and 349 cells/L and pre-treatment viral load between 100,000 and < 500,000 copies/mL. ^b^ Time since start of caRT. ^c^ Pre-treatment CD4 cell count (cells/L). ^d^ Pre-treatment viral load (copies/mL).

Appendix-table 2. Characteristics of the 6435 patients from the model-fitting dataset

|  | Pre-treatment HIV-1 RNA (copies/mL) | | | |  |
| --- | --- | --- | --- | --- | --- |
|  | <10k | 10k to <100k | 100k to <500k | 500k |  |
| Number of patients | 520 | 2227 | 2599 | 1089 |  |
| Median (IQR)^a^ age (years) | 37 (31-43) | 37 (31-43) | 37 (32-43) | 38 (32-45) |  |
| Male % | 56 | 73 | 78 | 79 |  |
| Risk group % |  |  |  |  |  |
| Homo/bisexual | 34 | 54 | 61 | 59 |  |
| IDU | 4 | 3 | 2 | 2 |  |
| Heterosexual | 56 | 38 | 33 | 34 |  |
| Other/not known | 6 | 5 | 4 | 5 |  |
| Ethnicity % |  |  |  |  |  |
| White | 40 | 56 | 60 | 62 |  |
| Black African | 43 | 27 | 23 | 24 |  |
| Other | 14 | 15 | 14 | 13 |  |
| Not known | 3 | 2 | 2 | 2 |  |
| First-line cART-regimen % |  |  |  |  |  |
| NNRTI | 54 | 63 | 66 | 63 |  |
| PI | 8 | 6 | 5 | 5 |  |
| Boosted-PI | 32 | 26 | 24 | 27 |  |
| Other | 6 | 5 | 5 | 6 |  |
| Median (IQR) pre-treatment  HIV-1 RNA (log_10_ copies/ml) | 3.41  (2.81-3.79) | 4.67  (4.43-4.86) | 5.32  (5.16-5.51) | 5.88  (5.71-6.01) |  |
| Median (IQR) pre-treatment  CD4 cell count (cells/L) | 270  (165-400) | 231  (150-320) | 180  (80-268) | 110  (43-207) |  |

^a^ IQR: Inter-quartile range; ^b^ k: A thousand

Appendix Table 3. Characteristics of the 3127 patients from the validation dataset.

|  | Pre-treatment HIV-1 RNA (copies/mL) | | | |
| --- | --- | --- | --- | --- |
|  | <10k | 10k to <100k | 100k to <500k | 500k |
| Number of patients | 236 | 1145 | 1226 | 520 |
| Median (IQR)^a^ age (years) | 36 (31-41) | 36 (31-43) | 38 (32-44) | 39 (33-45) |
| Male % | 58 | 75 | 82 | 81 |
| Risk group % |  |  |  |  |
| Homo/bisexual | 38 | 58 | 63 | 59 |
| IDU | 3 | 2 | 2 | 2 |
| Heterosexual | 51 | 36 | 31 | 36 |
| Other/not known | 7 | 5 | 4 | 3 |
| Ethnicity % |  |  |  |  |
| White | 41 | 58 | 61 | 62 |
| Black African | 44 | 28 | 23 | 26 |
| Other | 14 | 13 | 15 | 12 |
| Not known | 1 | 1 | 2 | 1 |
| First-line cART-regimen % |  |  |  |  |
| NNRTI | 50 | 63 | 69 | 63 |
| PI | 6 | 5 | 5 | 5 |
| Boosted-PI | 36 | 28 | 22 | 28 |
| Other | 8 | 4 | 4 | 4 |
| Median (IQR) pre-treatment  HIV-1 RNA (log_10_ copies/ml) | 3.51  (2.94-3.78) | 4.68  (4.43-4.87) | 5.31  (5.15-5.48) | 5.86  (5.71-5.99) |
| Median (IQR) pre-treatment  CD4 cell count (cells/L) | 276  (196-391) | 242  (168-321) | 187  (90-277) | 122  (40-242) |

^a^ IQR: Inter-quartile range; ^b^ k: A thousand

Appendix Table 4. Sensitivity analysis regarding observed and predicted suppression defined respectively by two consecutive observed and predicted viral load measurements 200 copies/mL. Validation of the model for predicting future suppression by 6 months since start of treatment given observations up to a specified visit.

|  | 2-month visit | 3-month visit | 4-month visit |
| --- | --- | --- | --- |
| No. patients^$^ | 2787 | 2224 | 1782 |
| Observed suppressed | 57% | 51% | 43% |
| Predicted suppressed | 59% | 51% | 42% |
| Sensitivity [95% CI^#^] | 90% [89%, 92%] | 93% [92%, 95%] | 96% [94%, 97%] |
| Specificity [95% CI] | 82% [80%, 84%] | 93% [92%, 95%] | 96% [94%, 97%] |
| PPV [95% CI] | 87% [85%, 89%] | 93% [92%, 95%] | 94% [92%, 96%] |
| NPV [95% CI] | 86% [84%, 88%] | 93% [91%, 94%] | 97% [96%, 98%] |
| LR+ [95% CI] | 5.03 [4.45, 5.68] | 13.37 [10.76, 16.61] | 21.40 [16.13, 28.39] |
| LR– [95% CI] | 0.12 [0.10, 0.14] | 0.07 [0.06, 0.09] | 0.04 [0.03, 0.06] |
| DOR [95% CI] | 42.16  [33.77, 52.62] | 183.15  [131.88, 254.36] | 481.66  [303.67, 763.98] |

$ Number of patients not suppressed at the specified visit and with at least one future measurement.

Abbreviations: CI is confidence interval; PPV is positive predictive value; NPV is negative predictive value; LR+ is likelihood ratio of a positive result; LR– is likelihood ratio of a negative result; DOR is diagnostic odds-ratio.

Appendix Table 5. Sensitivity analysis regarding the validation dataset was a random sample of the entire analysis dataset. Validation of the model for predicting future suppression by 6 months since start of treatment given observations up to a specified visit.

|  | 2-month visit | 3-month visit | 4-month visit |
| --- | --- | --- | --- |
| No. patients^$^ | 1486 | 872 | 532 |
| Observed suppressed | 78% | 67% | 49% |
| Predicted suppressed | 79% | 66% | 50% |
| Sensitivity [95% CI^#^] | 85% [83%, 87%] | 81% [77%, 84%] | 77% [72%, 82%] |
| Specificity [95% CI] | 45% [39%, 50%] | 63% [57%, 69%] | 76% [71%, 81%] |
| PPV [95% CI] | 84% [82%, 86%] | 81% [78%, 85%] | 76% [71%, 81%] |
| NPV [95% CI] | 47% [41%, 52%] | 62% [56%, 67%] | 78% [73%, 83%] |
| LR+ [95% CI] | 1.54 [1.40, 1.71] | 2.18 [1.86, 2.54] | 3.26 [2.60, 4.08] |
| LR– [95% CI] | 0.33 [0.27, 0.39] | 0.31 [0.26, 0.37] | 0.30 [0.24, 0.38] |
| DOR [95% CI] | 4.71 [3.60, 6.18] | 7.07 [5.16, 9.69] | 10.84 [7.25, 16.20] |

$ Number of patients not suppressed at the specified visit and with at least one future measurement.

Abbreviations: CI is confidence interval; PPV is positive predictive value; NPV is negative predictive value; LR+ is likelihood ratio of a positive result; LR– is likelihood ratio of a negative result; DOR is diagnostic odds-ratio.

Appendix Table 6. Sensitivity analysis regarding observations were not censored after the end of first-line cART. Validation of the model for predicting future suppression by 6 months since start of treatment given observations up to a specified visit

|  | 2-month visit | 3-month visit | 4-month visit |
| --- | --- | --- | --- |
| No. patients^$^ | 1933 | 1134 | 707 |
| Observed suppressed | 81% | 68% | 50% |
| Predicted suppressed | 75% | 65% | 51% |
| Sensitivity [95% CI^#^] | 82% [80%, 83%] | 79% [76%, 82%] | 79% [75%, 83%] |
| Specificity [95% CI] | 52% [47%, 57%] | 65% [60%, 70%] | 79% [74%, 83%] |
| PPV [95% CI] | 88% [86%, 89%] | 83% [80%, 86%] | 79% [75%, 83%] |
| NPV [95% CI] | 41% [36%, 45%] | 59% [54%, 64%] | 79% [75%, 83%] |
| LR+ [95% CI] | 1.70 [1.53, 1.90] | 2.26 [1.96, 2.62] | 3.70 [3.01, 4.55] |
| LR– [95% CI] | 0.35 [0.31, 0.41] | 0.32 [0.28, 0.38] | 0.26 [0.21, 0.33] |
| DOR [95% CI] | 4.82 [3.79, 6.12] | 7.01 [5.31, 9.25] | 14.02 [9.77, 20.13] |

$ Number of patients not suppressed at the specified visit and with at least one future measurement.

Abbreviations: CI is confidence interval; PPV is positive predictive value; NPV is negative predictive value; LR+ is likelihood ratio of a positive result; LR– is likelihood ratio of a negative result; DOR is diagnostic odds-ratio.

Appendix Table 7: Sensitivity analysis regarding censoring of first suppressed measurements below the detection of limit. Validation of the model for predicting future suppression by 6 months since start of treatment given observations up to a specified visit

|  | 2-month visit | 3-month visit | 4-month visit |
| --- | --- | --- | --- |
| No. patients^$^ | 1237 | 652 | 393 |
| Observed suppressed | 77% | 61% | 40% |
| Predicted suppressed | 77% | 63% | 45% |
| Sensitivity [95% CI^#^] | 83% [81%, 86%] | 77% [73%, 82%] | 78% [71%, 84%] |
| Specificity [95% CI] | 46% [40%, 52%] | 61% [55%, 67%] | 77% [72%, 83%] |
| PPV [95% CI] | 84% [81%, 86%] | 76% [72%, 80%] | 70% [63%, 77%] |
| NPV [95% CI] | 44% [39%, 50%] | 63% [57%, 69%] | 84% [79%, 89%] |
| LR+ [95% CI] | 1.53 [1.37, 1.71] | 1.99 [1.69, 2.35] | 3.45 [2.69, 4.44] |
| LR– [95% CI] | 0.37 [0.30, 0.45] | 0.37 [0.30, 0.45] | 0.29 [0.21, 0.39] |
| DOR [95% CI] | 4.16 [3.11, 5.55] | 5.41 [3.83, 7.64] | 12.07 [7.44, 19.59] |

$ Number of patients not suppressed at the specified visit and with at least one future measurement.

Abbreviations: CI is confidence interval; PPV is positive predictive value; NPV is negative predictive value; LR+ is likelihood ratio of a positive result; LR– is likelihood ratio of a negative result; DOR is diagnostic odds-ratio.

**Appendix References**

1. Taylor JMG and Law N. **Does the covariance structure matter in longitudinal modelling for the prediction of future CD4 counts?** *Stat Med* 1998; **17**: 2381–2394.
2. Diggle PJ, Heagerty P, Liang KY, and Zeger SL. *Analysis of Longitudinal Data, 2nd edition*. Oxford: Oxford University Press; 2002.
